# Executive Function and Self-Regulation: Bi-Directional Longitudinal Associations and Prediction of Early Academic Skills

**DOI:** 10.3389/fpsyg.2021.733328

**Published:** 2021-10-27

**Authors:** Steven J. Howard, Elena Vasseleu, Cathrine Neilsen-Hewett, Marc de Rosnay, Amy Y. C. Chan, Stuart Johnstone, Myrto Mavilidi, Fred Paas, Edward C. Melhuish

**Affiliations:** ^1^School of Education, University of Wollongong, Wollongong, NSW, Australia; ^2^School of Psychology, University of Wollongong, Wollongong, NSW, Australia; ^3^Early Start, University of Wollongong, Wollongong, NSW, Australia; ^4^Department of Psychology, Education and Child Studies, Erasmus University Rotterdam, Rotterdam, Netherlands; ^5^Department of Education, University of Oxford, Oxford, United Kingdom

**Keywords:** executive function, self-regulation, early childhood, preschool, academic, longitudinal

## Abstract

Despite a tendency to study executive function (EF) and self-regulation (SR) separately, parallel lines of research suggest considerable overlap between the two abilities. Specifically, both show similar developmental trajectories (i.e., develop rapidly in the early years), predict a broad range of overlapping outcomes across the lifespan (e.g., academic success, mental and physical health, and social competence), and have overlapping neural substrates (e.g., prefrontal cortex). While theoretical frameworks diverge in how they reconcile EF and SR – ranging from treating the two as functionally synonymous, to viewing them as related yet distinct abilities – there is no consensus and limited empirical evidence on the nature of their relationship and how this extends developmentally. The current study examined bi-directional longitudinal associations between early EF and SR, and their longitudinal associations with subsequent early academic skills, in a sample of 199 3- to 5-year-old pre-school children. The adopted measures permitted EF and SR to be modelled as composite indices for these analyses, thereby decreasing task-specific components of these associations. Early academic skills were captured by a standardized direct assessment. Bi-directional associations between EF and SR were found, with both accounting for unique variance in early academic skills 7 and 19months later. The current results provide important evidence to distinguish between EF and SR abilities, yet also for their reciprocal influence *in situ* and across early development.

## Introduction

Parallel but largely independent lines of research have established that early executive function (EF) and self-regulation (SR) abilities are influential to wide-ranging developmental trajectories and for later-life outcomes. EF and SR both develop rapidly in the early years of life and continuously into adolescence (Montroy et al., 2016), predict a broad range of overlapping outcomes across the lifespan (e.g., academic success, mental health, physical health, and social competence; Howard and Williams, 2018), and have overlapping neural substrates (e.g., prefrontal cortex; Cohen and Lieberman, 2010). Inter-task correlations are often found between EF and SR measures (Howard and Melhuish, 2017); indeed, these constructs are sometimes functionally conflated, such that single measures have been separately construed as indexing SR or EF (e.g., McClelland et al., 2014). Furthermore, interventions that target one of these abilities often also evaluate impact on the other, given theoretical and empirical accounts of EF-SR associations and expectations for commensurate growth (e.g., Domitrovich et al., 2007).

Yet, there is also evidence that EF and SR should be treated as functionally distinct. Despite frequent expectations to the contrary, effects of interventions targeting one ability infrequently transfer to the other (near transfer to untrained applications of the trained ability is also less common; Kassai et al., 2019). This is perhaps to be expected given that modest cross-sectional associations between EF and SR are typically observed (although this may be a function of measurement imprecision; Blair et al., 2005; Carlson, 2005), and theoretical models that imply different mechanisms for the application of these abilities (although there are also models that attempt to integrate the two abilities in explaining behavior; Hofmann et al., 2012). Lack of clarity about the nature of the relationship between EF and SR in development is exacerbated by relatively poor consensus on definition, delineation, and measurement of these constructs, which makes it difficult to generate and test explicit models that integrate the development of EF and SR. Yet, this knowledge gap provides the impetus to identify and instigate models of EF and SR change, and thereby realise the short- and long-term benefits that have been speculated as a consequence of growth in these foundational abilities (Moffitt et al., 2011). Alternatively, integrated models of EF and SR development may clarify the nuanced ways in which they have independent or shared influences on outcomes. The current study sought to provide some insight into this issue, investigating the longitudinal bi-directional associations between early childhood EF and SR, as well as their independent and cumulative prediction of early academic skills in the first year of school.

### Definitions and Delineations of EF and SR

One complication in expounding an integrated model of EF and SR growth is diversity in conceptions, definitions, and operationalisations of key constructs and the relations between them. For instance, EF is generally considered a collection of cognitive control capacities to activate and maintain mental information (working memory), resist contrary urges (inhibition), and flexibly shift attention (cognitive flexibility; Miyake et al., 2000). While the number and organisation of EFs are debated (Lehto et al., 2003), a prominent taxonomy distinguishes between these three core EFs, which are the basis for more complex, higher order cognition (Miyake et al., 2000). Further complicating this picture, there is evidence that the ability to disaggregate these EFs changes over the preschool to early primary school years (Zelazo et al., 2013).

Even greater diversity exists in conceptualizations of SR (Burman et al., 2015), although definitions tend to emphasize its role in exerting control over manifest behaviors, emotional reactions, and social interactions (some conceptions term this “self-control,” due to the need to override unwanted impulses; Hofmann et al., 2012). Whereas EFs enable control over *mental activity*, SR has been distinguished as enabling control over *in situ*, and often emotionally laden *manifest responses*. This is not to say that SR applies solely to the control of emotions; indeed, factor analysis of SR measures often identifies separate emotional, cognitive, and behavioral SR factors (e.g., Howard and Melhiush, 2017; Howard et al., 2019). Whereas cognitive SR is often considered to be concerned with attentional and higher order cognitive control (Blair, 2016), behavioral SR is often used to describe children’s ability to control their actions in everyday contexts (Howard and Melhiush, 2017). In early childhood, SR thus has broad and multiple applications such as waiting one’s turn despite the impulse to act now, overcoming strong emotions in order to respond adaptively, and remaining within the rules and requirements of the setting (e.g., at preschool vs. home).

Despite conceptual overlap between EF and SR, the extent to which they are related and the nature, mechanisms, and shifts in such relations across childhood remain unclear. For instance, one model to explain self-regulatory success posits EFs as the capacity to overcome obstacles and contrary impulses to reduce a discrepancy between actual and goal states (Baumeister and Heatherton, 1996). In this model, EFs are necessary but not sufficient for successful SR. Also essential are selection and maintenance of goals, and motivation to continually invest effort until goal achievement. By contrast, in the bi-directional model of EF and SR (Blair and Ursache, 2011; Blair, 2016) EFs are top-down mechanisms by which an individual can direct attention and manage arousal (Ochsner and Gross, 2005) for the purposes of goal-directed action. From a bottom-up perspective, the mobilisation of EFs is influenced by activity in stress, emotional, and attentional systems (Blair and Dennis, 2010). That is to say, within this model EFs are necessary for successful SR but may be impaired by particularly high or low levels of arousal (i.e., reactivity). In this sense, the capacity for SR maps well on to inverted U-shaped Yerkes-Dodson curve, wherein EF proficiency is maximized at moderate levels of emotional and attentional reactivity, but is undermined at overly high or low reactivity levels (Arnsten, 2009).

While both models described above envision an interaction between EF and SR, the bi-directional model uniquely includes mechanisms by which EF and SR may be mutually influential throughout development (Blair, 2016). For example, exposure to chronic stress – requiring frequent and effortful SR – can release neurochemicals affecting activity and development of the prefrontal cortex, thereby influencing EF development (Cerqueira et al., 2007; Liston et al., 2009). Reciprocally, children with higher EFs have a wider allostatic range in which they can self-regulate under conditions of heightened arousal, and thus can engage and extend their SR abilities across a wider range of challenge. The bi-directional model, which implies that causal influences on development stem from both EF and SR processes, has important implications for identifying plausible targets for education, prevention, and intervention. For example, this model requires us to ask whether EF should in fact be targeted (as is common) to achieve real-world SR benefits; the possibility at least needs to be examined that SR change should be addressed directly and, furthermore, whether such changes in SR produce EF benefits. In sum, the bi-directional model potentiates greater clarity regarding how EF and SR develop, and how the relationship between them underpins important features of later development (i.e., academic success, mental health, physical health, and social competence).

### Implications for School Readiness and Success

The area where EF and SR growth have received the most attention is in relation to school readiness and academic success. Independent investigation of links between EFs and academic achievement show preschool EF abilities accounting for substantial variability in later academic achievement (Welsh et al., 2010; Fuhs et al., 2014). For instance, Bull et al. (2008) showed that high preschool EF provided an immediate head start in mathematics and reading that persisted across the first 3years of schooling. This association is robust, with numerous studies showing that relationships between EF and later academic achievement (i.e., mathematics and reading) remain even after controlling for general cognitive abilities (Espy et al., 2004; Clark et al., 2010), baseline measures of academic ability (McClelland et al., 2007), and general intelligence (Fitzpatrick et al., 2014). Furthermore, there is some evidence that this sequence is causal, with Ribner et al. (2017) showing that children with better EFs were able to catch up to peers who initially had better early mathematics abilities. Similarly, SR abilities in preschool have also been independently linked with school readiness and later academic achievement. For instance, early SR predicts status and change in academic abilities (i.e., literacy, vocabulary, and mathematics skills) across the final preschool year (McClelland et al., 2007) and during the transition to school (McClelland and Wanless, 2012). Self-regulation at age 4 has also been linked with academic achievement at age 7 (i.e., in mathematics and reading) with effects persisting into early adulthood (McClelland et al., 2013).

To explain these associations, researchers have proposed distinct and complementary ways in which EF and SR may influence academic achievement. With regard to the effects of EF on academic achievement, researchers have emphasized the direct facilitative role EFs play in learning (e.g., to remain focused, hold information in the mind, resist distraction), as well as specific links between EF processes and the inherent requirements of learning tasks (Blair et al., 2015). By contrast, proposed mechanisms for the direct impact of SR on academic achievement have tended to focus on broader contextual determinants of engagement in education. For example, given that lower SR is linked with poorer teacher–child relationships and heightened conflict (Hamre and Pianta, 2001; Valiente et al., 2011), SR may support or constrain adaptive engagement with learning environments and/or with educators more directly.

These explanations are consistent with Blair and Raver’s (2015) extension of the bidirectional model of EF and SR to school readiness, in which children are ready to start school (i.e., they are well-positioned to benefit from its structures for teaching and learning) when they are sufficiently able to regulate their arousal and attention to sustain engagement with learning experiences. While efforts have been made to independently link EFs and SR to school readiness, limited empirical research investigates the longitudinal and potentially bi-directional associations between EF and SR, and their unique/shared prediction of important developmental outcomes. Yet these insights are important for establishing a developmental model that integrates EF and SR, and for theoretical models of change that underpin EF and/or SR growth.

### The Current Study

The current study thus sought – in a sample of preschool-aged children assessed three times over nearly 2years – to investigate the bidirectional relationships between EF and SR longitudinally, as well as the independent and cumulative associations of EF and SR with school readiness over the transition to school period. In line with predictions of Blair and colleagues’ bi-directional model (Blair and Ursache, 2011; Blair, 2016), it was expected that EF and SR would be distinct yet related, with bi-directional associations due to their theorized reciprocal influence. Further, it was expected that EF and SR would independently and cumulatively predict early academic skills on entry to school, given their expected unique contributions to engagement in and acquisition during early learning experiences. If supported, such a model would suggest potential benefit for an integrated approach to promoting EF and SR growth.

## Materials and Methods

### Design and Participants

This was a longitudinal observational study of EF, SR, and academic skills with data collected at three time points: T_1_ at the beginning of children’s final preschool year; T_2_ at end of the final preschool year; and T_3_ 1-year later, at the end of the first year of school. It leveraged data from a cluster randomized controlled trial (RCT) intervention evaluation (Howard et al., 2020), collecting data at a third time point from a geographically constrained subset of participating children who had transitioned to formal school in the following year. Longitudinal studies tracking children between preschool and school in Australia are complex because there is relatively little continuity between preschool and school contexts; children from a single preschool often disperse to many geographically distributed schools (up to nine, in the current sample) depending on jurisdiction. For this reason, a geographically defined constraint was used when recruiting children for T_3_ data collection. The characteristics of the geographically constrained sample are described in detail below. The initial RCT (see Howard et al., 2020 for a description) revealed little difference between groups, which justified collapsing across groups for the purposes of the current study. Nevertheless, possible longitudinal impacts of the intervention were examined in relation to key T_3_ measures to further ensure this strategy was justified, analyses of which showed no significant difference between groups on any modelled T_3_ measure.

Children who had transitioned to 160 schools within the geographic radius were eligible and invited to participate in the follow-up, yielding an eligible sample of 316 children. Of these children, data were collected at T_3_ (i.e., end of first year of formal schooling) for 199 children. Reasons for non-participation at this time point were parental non-consent (*n*=53 unable to contact, *n*=31 declined) or schools declining participation (impacting participation of *n*=33 children). The mean age of the final sample, based on baseline demographics, was 4.46years (*SD*=0.35, range 3.65–5.24), with 51.8% girls. Children who were identified as of Aboriginal or Torres Strait Islander descent comprised 3.0% of the sample, which is in line with population estimates for this age (Australian Institute of Health and Welfare, 2012). Family income was diverse: 6.5% of families qualified for full childcare benefit subsidies (low income); 53.3% of families qualified for some childcare benefit subsidy (low-middle to middle-high income); 23.6% of families did not qualify for any childcare benefit subsidy (very high income); while 16.6% of families declined to disclose. The highest level of maternal education attained was also diverse: 6.0% did not complete high school; 8.0% completed high school; 22.1% had completed diploma, trade, certificate; 35.2% completed a tertiary degree; 12.6% a post-graduate qualification; and 16.1% did not disclose. The 112 participating schools spanned Public (*n*=82), Catholic (*n*=28) and Independent School systems (*n*=2). The mean number of children per participating school was 1.78 (*SD*=1.21, range=1–8). These children derived from 41 preschool services, which were diverse in their socio-economic decile for catchment area (*M*=6.63, *SD*=2.44, range=1–10) and statutory quality assessment rating (i.e., *n*=20 Exceeding, *n*=19 Meeting, *n*=1 Working Toward, *n*=1 unrated against the National Quality Standard).

This study was approved by the University’s Human Research Ethics Committee, Social Sciences (2018/536), and participants were those who provided verbal assent and their parents provided informed written consent to participate.

### Measures

#### Self-Regulation

The *Head-Toes-Knees-Shoulder* task (HTKS; McClelland et al., 2014) asks children to remember a correspondence between body parts (e.g., head and knees), and then perform the opposite action to what was indicated (e.g., touch their knees when the facilitator says “touch your head”). The task consists of six practice and 10 test trials at each of three levels: (1) correspondence between head and toes; (2) correspondence between knees-shoulders and head-toes; and (3) flexibly switching between the correspondences of head-knees and shoulders-toes. The task continues until completion or failure to achieve at least four points within a level (such that two points are awarded for a correct response and one point for a self-corrected correct response). This task takes an average of ~6min to complete. Performance was indexed by the sum of points awarded for all practice and test trials attempted, yielding a score with a possible range from 0 to 94. Reliability in the current study was similarly strong (T_1_ *α*=0.97, T_2_ *α*=0.97) to previous reports (McClelland et al., 2014).

The *Preschool Situational Self-Regulation Toolkit Assessment* (PRSIST; Howard et al., 2019) is an observational measure of early SR that engages children in self-regulatory activities and rates the child’s behavior in relation to cognitive and behavioral self-regulation. The first PRSIST Assessment activity is a memory card game. In this activity, children in a group of four take turns trying to find a matching pair of cards (e.g., eight pairs for 4-year olds, 14 pairs for 5-year olds), taking around 10min to complete. The second activity is an individual curiosity boxes’ activity, in which children are presented with a series of three boxes of increasing size and are asked to guess their contents. The sequence of guessing occurs as follows: first, guess based only on the size of the box (no touching); second, guess after gently lifting the box to feel its weight (no shaking); third, guess after shaking the box (no opening); and lastly, guess after closing your eyes and feeling the object inside (no peeking). This activity takes approximately 5min to complete. Each child’s self-regulation was rated at the end of each activity. Items were scored along a seven-point Likert scale, with the ratings representing a judgement of the frequency and/or severity of behaviors pertaining to cognitive self-regulation (e.g., did the child sustain attention, and resist distraction, during the instructions and activity?) and behavioral self-regulation (e.g., did the child control their behaviors and stay within the rules of the activity?). This yielded two sets of ratings per child, which were averaged for the two activities before aggregating into cognitive (six items) and behavioral self-regulation indices (three items) with a possible range from 1 to 7. Reliability in the current study was similarly strong (T_1_ *α*=0.92, T_2_ *α*=0.90) in line with previous reports (Howard et al., 2019).

Educator-reports of children’s self-regulation on the *Child Self-Regulation and Behavior Questionnaire* (CSBQ; Howard and Melhuish, 2017) were also collected. This scale consists of 34 items pertaining to the typicality of children’s everyday behaviors (e.g., “Persists with difficult tasks”). Each item was rated by the child’s educator along a five-point Likert scale from “Not true” to “Certainly true” about the child. Ratings on individual items were averaged to generate subscales of cognitive (five items), behavioral (six items) and emotional self-regulation (six items), as well as subscales concerning prosocial behavior, sociability, internalising problems and externalising problems, with a possible range from 1 to 5. Reliability in the current study was similarly strong (T_1_: cognitive *α*=0.87, behavioral *α*=0.88, emotional *α*=0.79; T_2_: cognitive *α*=0.89, behavioral *α*=0.87, emotional *α*=0.85) to previous reports (Howard and Melhuish, 2017).

#### Executive Functions

The three core EFs were assessed using assessments from the Early Years Toolbox (EYT), performed on iPads (Howard and Melhuish, 2017). Working memory was indexed by the *Mr. Ant* task, which asks children to remember the spatial locations of “stickers” placed on a cartoon ant and identify these locations after a brief retention interval. Test trials increase in complexity as the task progresses (progressing from one to eight stickers), with three trials at each level, until the earlier of completion of the task or failure on three trials at the same level of difficulty. Working memory was indexed by a point score that estimates working memory capacity, calculated as: one point for each level, from the first, in which at least two of three trials are performed correctly; and then one-third of a point for each correct trial thereafter (yielding a possible range from 0 to 8; Howard and Melhuish, 2017).

Inhibition was assessed by the *go/no-go* task, which requires participants to respond to “go” trials (“catch fish”) and withhold responding on the “no-go” trials (“avoid sharks”). The majority of stimuli are “go” trials (80% fish), thereby generating a pre-potent tendency to respond that children must inhibit on “no-go” trials (20% sharks). After instruction and practice, 75 test stimuli were presented across three 1-min blocks (separated by a short break and reiteration of instructions). Each trial involved presentation of an animated stimulus (i.e., fish or shark) for 1,500ms, each separated by a 1,000ms inter-stimulus interval. Inhibition was indexed by an impulse control score, which is the product of proportional “go” (to account for the strength of the pre-potent response generated) and “no-go” accuracy (to index a participant’s ability to overcome this pre-potent response), to yield a proportional accuracy score that ranged from 0.00 to 1.00.

Cognitive flexibility was assessed by the *Card Sort* task, which asks children to sort cards (i.e., red rabbits, blue boats) first by one sorting dimension (e.g., color), then switch to the other sorting dimension (e.g., shape). The task begins with a demonstration and two practice trials, after which children begin sorting by one dimension for six trials. In the subsequent post-switch phase, children are asked to switch to the other sorting dimension. For all test items, each trial begins by reiterating the relevant sorting rule and then presenting a stimulus for sorting. If the participant correctly sorts at least five of the six pre- and post-switch stimuli, they then proceed to a border phase of the task. In this phase, children are required to sort by color if the card has a black border or sort by shape if the card has no black border. Cognitive flexibility was indexed by the number of correct sorts after the pre-switch phase (yielding a score that ranged from 0 to 12; Howard and Melhuish, 2017). Inter-task correlations between EF measures in the current sample (*r*s from 0.16 to 0.30) were similar to those previously reported (Howard and Melhuish, 2017).

#### Early Academic Skills

Early academic knowledge of participating children was assessed at T_3_ using two measures from the EYT: Early Numeracy and Expressive Vocabulary 2. *EYT Early Numeracy* is an iPad-based assessment of young children’s early numeracy (Howard et al., 2021). It consists of 79 interspersed items pertaining to foundational domains (and subdomains) of early numeracy knowledge, including number sense, cardinality and counting, numerical operations, and special and measurement constructs. The assessment is administered *via* an iPad app, in which a child helps a cartoon robot solve the numerical problems it encounters. Sequencing of items, audio instructions and scoring are all managed by the app to standardize administration of the tool. Items are presented in consistent order of increasing difficulty. The app also has automated start rules based on age of the child, and a stop rule after five consecutive incorrect responses, yielding a mean administration time of ~7min. A total raw accuracy score indexes early numeracy performance, with a possible range of 0 to 79.

*EYT Expressive Vocabulary 2* is a 54-item measure of a child’s expressive vocabulary development (Howard and Melhiush, 2017). It requires children to verbally produce the correct label for a depicted stimulus (depicted noun or animated verb), which a data collector records within the app. In cases of an incorrect label initially being produced, the data collector prompts participants by asking “what else might this be called” until either a correct production or some indication that the child is unable to produce the required word. A six-item stop rule minimizes administration time to ~6min. An overall accuracy score indexes expressive vocabulary performance, with a possible range of 0 to 54.

#### Demographic Covariates

Parents reported on demographic information used as covariates for analyses. These were: child’s age (the date of assessment minus date of birth); child’s sex (1=male, 2=female); and a postcode-level index of socioeconomic decile created by the Australian Bureau of Statistics (i.e., the socioeconomic indexes for areas, SEIFA; Australian Bureau of Statistics, 2012), combining census data on factors such as education, household income, and unemployment.

### Procedure

At T_1_ and T_2_, all tasks were administered to children in a quiet area of their preschool centre in five sessions across the same day, to maximize children’s attention and minimize fatigue. Measures were administered in the same order to all children, as follows: (1) Bracken School Readiness Assessment (not used for the purposes of this analysis, given its use only at earlier time points and performance near ceiling by T_2_); (2) PRSIST curiosity boxes and HTKS; (3) Mr. Ant and Go/No-Go; (4) PRSIST memory; and (5) Card Sort. Each session took 10–20min to complete. For T_3_ data collection in schools, data were collected in a quiet space (e.g., office, library) across three sessions in the same day, as follows: (1) Mr. Ant and Go/No-Go; (2) Card Sort and HTKS; and (3) Numeracy, Expressive Vocabulary and Shape Trail (the latter not used for purposes of this study). Children’s Kindergarten teachers also reported on children’s SR using the CSBQ at the same time.

### Data Analysis

**Figure 1:**
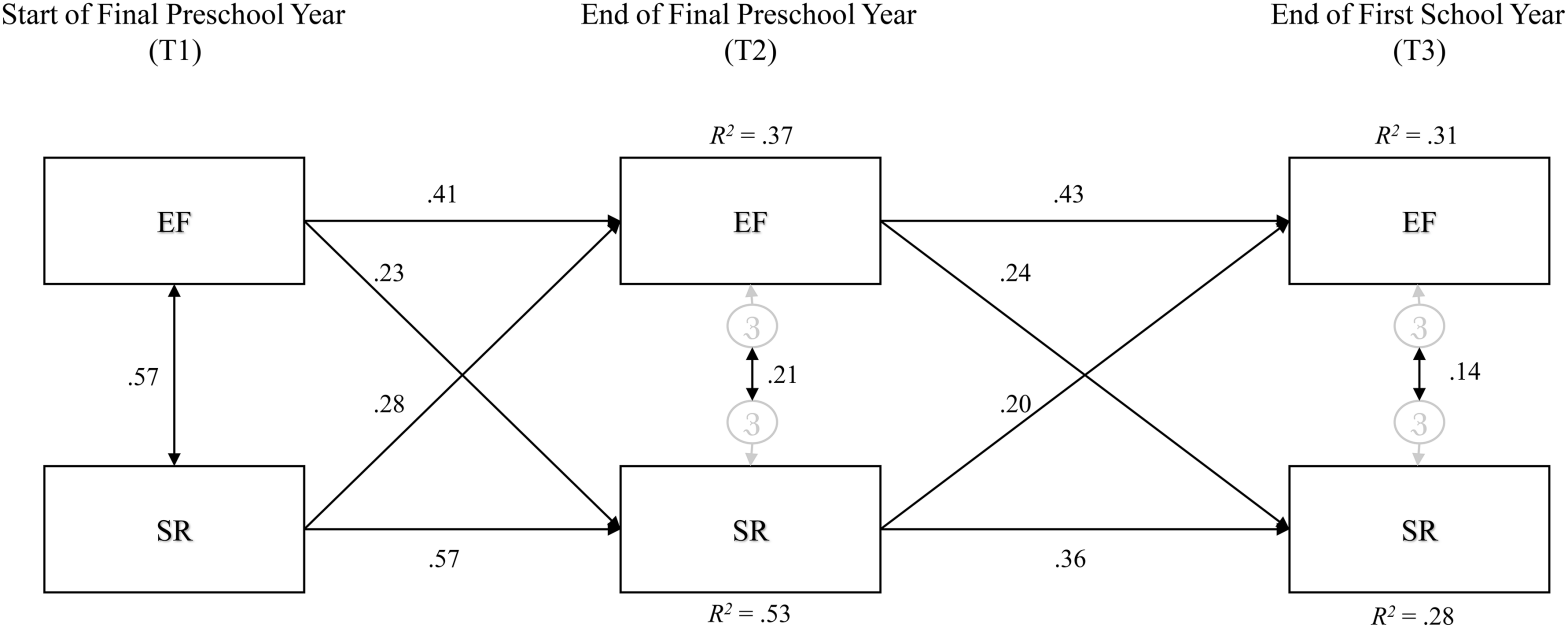
Model 1: cross-lagged panel model of EF and SR. Path loadings are standardized regression weights. EF, executive function composite index and SR, self-regulation composite index.

**Figure 2:**
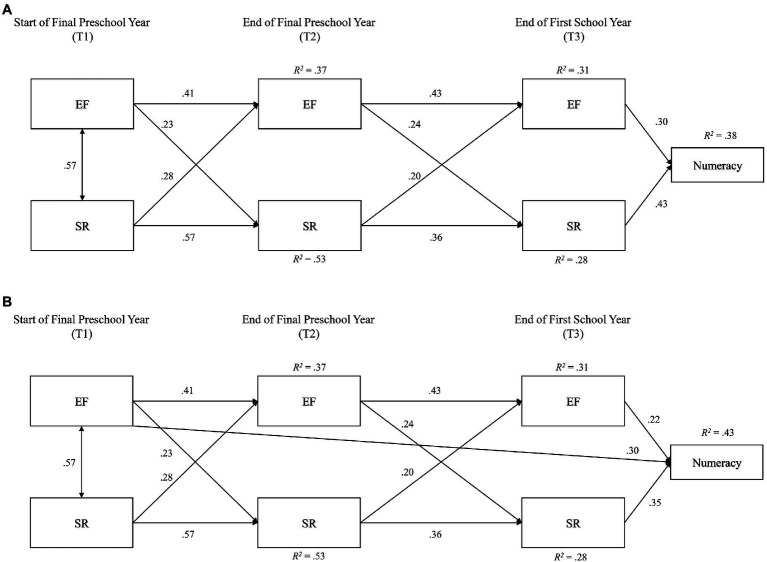
Model 2: **(A)** cross-lagged panel model predicting numeracy. Model 2: **(B)** cross-lagged panel model predicting numeracy, with direct path from T_1_ EF. Path loadings are standardized regression weights. Correlated error terms between T_2_ and T_3_ EF and SR were modelled but are omitted from this figure. EF, executive function composite index and SR, self-regulation composite index.

**Figure 3:**
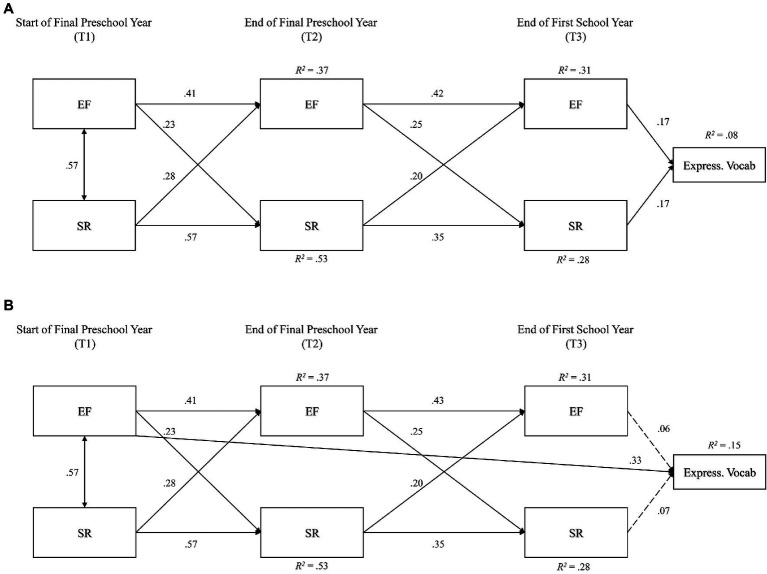
Model 3: **(A)** cross-lagged panel model predicting vocabulary. Model 3: **(B)** cross-lagged panel model predicting vocabulary, with direct path from T_1_ EF. Path loadings are standardized regression weights. Dashed lines are non-significant. Correlated error terms between T_2_ and T_3_ EF and SR were modelled but are omitted from this figure. EF, executive function composite index and SR, self-regulation composite index.

To evaluate the bi-directional associations between EF and SR, and longitudinal relations to early academic skills, cross-lagged panel models were run using AMOS (Version 25, IBM Corp Armonk NY, United States). In line with theoretical predictions of EF and SR abilities as distinct – i.e., they are reciprocally influential and independently account for unique variance in development and outcomes – three models were evaluated. These evaluated a three-timepoint (start and end of preschool year, end of first year of school) cross-lagged panel model (Model 1, Figure 1), which subsequently added early numeracy skill as the outcome (Model 2, Figure 2) or expressive vocabulary as the outcome (Model 3, Figure 3). Given PRSIST was not possible to conduct at T_3_ due to a limited number of participating students per school, the T_3_ SR variable was a composite of HTKS and two school-teacher-reported CSBQ subscales: behavioral self-regulation and cognitive self-regulation. The stability of the SR factor over time, as a consequence of this change, was also evaluated through these models.

## Results

Table 1 provides descriptive statistics for EF, SR, early academic skills, and demographic variables. Bivariate correlations for all modelled variables are provided at Tables 2, 3. Path modelling using AMOS maximum likelihood estimation was used to evaluate absolute and relative fit of cross-lagged panel models. Given debate on treating EF and related abilities as a latent variable in early childhood and longitudinal analyses (i.e., given the need, although unlikely satisfaction, for longitudinal measurement invariance) a composite variable approach was used to generate EF and SR indices, in line with recent recommendations (Camerota et al., 2020). Composite scores were exploratory-factor-analysis-derived factor (standard) scores. Absolute model fit was evaluated using *χ*^2^ statistics, and relative model fit was assessed using a combination of Bentler’s comparative fit index (CFI, with values >0.90 suggested to indicate good model fit; Smith and McMillan, 2001), root mean square error of approximation (RMSEA, with values <0.05 suggested to indicate good model fit; Browne and Cudeck, 1993), and Akaike’s information criterion (AIC, with comparatively lower values indicating better model fit). For all early academic outcome models, the inclusion of child age, sex and SEIFA loaded onto the outcome as control variables did not significantly change the pattern or strength of EF or SR paths, or the amount of variance in the outcome accounted for by paths in the model. However, it did substantially reduce overall model fit due to its failure to account for multiple and complex associations between modelled variables and these control variables (e.g., age with EF and SR, which were not the focus of the current analyses). Given that patterns of significance, strength of path loadings and *R*^2^ did not differ between the models, and a reduced statistical power due to some missingness in control variables, results for models without control variables are presented. Sensitivity analyses with models controlling for the grouping variable in the source study (intervention, control) and models using only control group data showed highly similar model fit, strength and significance of path loadings. As such, results using the full sample are reported.

**Table 1:** Descriptive statistics for modelled variables.

	Time 1		Time 2		Time 3	
	M (SD)	Range	M (SD)	Range	M (SD)	Range
SR Factor	0.00 (0.82)	−2.06-2.09	0.00 (0.87)	−2.64-1.83	0.00 (0.97)	−3.70-1.11
HTKS	21.50 (23.69)	0–87	42.34 (27.12)	0–90	67.25 (20.10)	2–94
PRSIST	3.74 (1.10)	1.00–6.40	4.33 (1.04)	1.57–6.55	-	-
CSBQ SR	3.72 (0.70)	1.70–5.00	3.90 (0.74)	1.70–5.00	-	-
CSBQ CSR	-	-	-	-	3.84 (1.04)	1.00–5.00
CSBQ BSR	-	-	-	-	4.08 (1.00)	1.00–5.00
EF Factor	0.00 (0.77)	−1.69-2.26	0.00 (0.74)	−2.80-1.98	0.00 (0.76)	−2.73-1.45
EYT Mr. Ant	1.48 (0.91)	0.00–4.33	1.90 (0.84)	0.00–4.33	2.60 (0.78)	0.67–5.00
EYT GNG	0.57 (0.20)	0.04–0.97	0.71 (0.19)	0.00–1.00	0.79 (0.16)	0.20–1.00
EYT CS	4.46 (4.14)	0–12	6.27 (3.99)	0–12	8.90 (2.07)	0–12
EYT numeracy	-	-	-	-	64.26 (9.92)	28–80
EYT vocab	-	-	-	-	39.54 (7.44)	0–52

HTKS, Head-Toes-Knees-Shoulders task; PRSIST, Preschool Situational Self-Regulation Toolkit; CSBQ, Child Self-Regulation and Behavior Questionnaire; SR, self-regulation; CSR, cognitive self-regulation; BSR, behavioral self-regulation; EYT, Early Years Toolbox; GNG, Go/No-Go; CS, Card Sort; and Vocab, expressive vocabulary.

**Table 2:** Bivariate correlations between composite indices.

		1	2	3	4	5	6	7	8	9	10	11	12	13	14	15	16	17	18
1	HTKS T1	-	0.59^*^	0.38^*^	0.41^*^	0.38^*^	0.32^*^	0.22^*^	0.36^*^	0.36^*^	0.35^*^	0.26^*^	0.21^*^	0.31^*^	0.24^*^	0.19^*^	0.42^*^	0.31^*^	0.20^*^
2	HTKS T2		-	0.52^*^	0.46^*^	0.46^*^	0.37^*^	0.26^*^	0.42^*^	0.36^*^	0.38^*^	0.34^*^	0.27^*^	0.33^*^	0.29^*^	0.25^*^	0.39^*^	0.34^*^	0.25^*^
3	HTKS T3			-	0.41^*^	0.33^*^	0.36^*^	0.23^*^	0.37^*^	0.27^*^	0.33^*^	0.41^*^	0.29^*^	0.31^*^	0.28^*^	0.35^*^	0.28^*^	0.31^*^	0.33^*^
4	PRSIST T1				-	0.53^*^	0.42^*^	0.38^*^	0.36^*^	0.36^*^	0.43^*^	0.35^*^	0.23^*^	0.37^*^	0.33^*^	0.36^*^	0.29^*^	0.30^*^	0.27^*^
5	PRSIST T2					-	0.44^*^	0.40^*^	0.39^*^	0.40^*^	0.37^*^	0.32^*^	0.21^*^	0.39^*^	0.40^*^	0.34^*^	0.32^*^	0.35^*^	0.31^*^
6	CSBQ T1						-	0.65^*^	0.44^*^	0.50^*^	0.27^*^	0.22^*^	0.14	0.37^*^	0.26^*^	0.29^*^	0.19^*^	0.19^*^	0.19^*^
7	CSBQ T2							-	0.35^*^	0.45^*^	0.27^*^	0.19^*^	0.06	0.33^*^	0.23^*^	0.29^*^	0.08	0.16^*^	0.29^*^
8	CBSQ-C T3								-	0.68^*^	0.26^*^	0.35^*^	0.25^*^	0.37^*^	0.31^*^	0.30^*^	0.29^*^	0.26^*^	0.31^*^
9	CSBQ-B T3									-	0.27^*^	0.33^*^	0.25^*^	0.40^*^	0.31^*^	0.30^*^	0.26^*^	0.14	0.16*
10	MrAnt T1										-	0.41^*^	0.31^*^	0.30^*^	0.33^*^	0.26^*^	0.28^*^	0.25^*^	0.16^*^
11	MrAnt T2											-	0.47^*^	0.28^*^	0.36^*^	0.38^*^	0.18^*^	0.20^*^	0.12
12	MrAnt T3												-	0.25^*^	0.23^*^	0.31^*^	0.13	0.14	0.13
13	GNG T1													-	0.42^*^	0.40^*^	0.16^*^	0.19^*^	0.21^*^
14	GNG T2														-	0.36^*^	0.17^*^	0.20^*^	0.27^*^
15	GNG T3															-	0.11	0.17^*^	0.23^*^
16	Card Sort T1																-	0.43^*^	0.17^*^
17	Card Sort T2																	-	0.26^*^
18	Card Sort T3																		-

HTKS, Head-Toes-Knees-Shoulders task; PRSIST, Preschool Situational Self-Regulation Toolkit; CSBQ, Child Self-Regulation and Behavior Questionnaire; C, cognitive self-regulation; B, behavioral self-regulation; GNG, Go/No-Go; T1, Time 1; T2, Time 2; and T3, Time 3.

* *p*<0.05.

**Table 3:** Bivariate correlations between modelled variables.

		1	2	3	4	5	6	7	8
1	SR T1	-	0.70^*^	0.52^*^	0.57^*^	0.49^*^	0.43^*^	0.52^*^	0.21^*^
2	SR T2		-	0.48^*^	0.53^*^	0.52^*^	0.44^*^	0.45^*^	0.27^*^
3	SR T3			-	0.39^*^	0.43^*^	0.39^*^	0.54^*^	0.25^*^
4	EF T1				-	0.55^*^	0.42^*^	0.52^*^	0.38^*^
5	EF T2					-	0.53^*^	0.46^*^	0.38^*^
6	EF T3						-	0.47^*^	0.24^*^
7	Numeracy T3							-	0.49^*^
8	Vocab T3								-

SR, self-regulation; EF, executive function; and Vocab, expressive vocabulary.

* *p*<0.05.

### Model 1: Three-Timepoint Cross-Lagged Panel Model

The three-timepoint model (Figure 1) provided good fit to the data: *χ*^2^(4)=13.42, *p*=0.009, CFI=0.99, RMSEA=0.07, AIC=59.42. Each of EF and SR showed moderate prediction of that same ability from each time point to the next (*βs* ranging from 0.36 to 0.57). Patterns of bi-directional association were also evident: EF and SR at T_1_ were moderately correlated (*r*=0.57) and there were small and similar loadings from EF to SR, and from SR to EF, from one time point to the next (*βs* ranging from 0.20 to 0.28). Lower correlations between EF and SR at later time points are to be expected due to variance accounted for by autoregressive paths – that is, each subsequent correlation between EF and SR accounts for prior levels of these abilities – and thus should not be interpreted as point-in-time reductions in correlation. As such, they are not further interpreted or presented in depictions of subsequent models.

### Model 2: Three-Timepoint Cross-Lagged Panel Model Predicting Numeracy

The addition of early numeracy as an outcome showed slightly reduced, albeit still good, model fit: *χ*^2^(8)=41.38, *p*<0.001, CFI=0.96, RMSEA=0.09, AIC=95.38. In terms of prediction of early academic skills, EF (*β*=0.30) and SR (*β*=0.43) at T_3_, both independently predicted numeracy scores (Figure 2A). Constraining these paths to equivalence did not significantly alter model fit, which suggests that the path loadings onto early numeracy were comparable. *R*^2^ statistics indicated that this model accounted for: at T_3_, 38% of the variance in early numeracy skills, 31% of the variance in EF and 28% of the variance in SR; and at T_2_, 37% of the variance in EF and 53% of the variance in SR. Modification indices suggested model improvement with inclusion of additional paths from T_1_ and T_2_ EF and SR to T_3_ numeracy skills, which were added sequentially by order of their strength and evaluated in Model 2b.

The final revised model (Figure 2B) provided better relative fit to the data: *χ*^2^(7)=19.28, *p*=0.007, CFI=0.99, RMSEA=0.07, AIC=76.33. While all paths from the initial Model 2 remained significant, there was an additional significant path from T_1_ EF to T_3_ numeracy (*β*=0.30), which slightly reduced the strength of paths from T_3_ EF (*β*=0.22) and SR to numeracy (*β*=0.35). This model provided better explanation of early numeracy as well, *R*^2^=0.43. No further modification indices were indicated after addition of this path.

### Model 3: Three-Timepoint Cross-Lagged Panel Model Predicting Expressive Vocabulary

The three-timepoint model integrating expressive vocabulary (Figure 3A) also provided good fit to the data: *χ*^2^(8)=37.99, *p*<0.001, CFI=0.97, RMSEA=0.09, AIC=91.99. In terms of prediction of early academic skills, EF (*β*=0.17) and SR (*β*=0.17) at T_3_ both equally and independently predicted expressive vocabulary scores (Figure 2A). *R*^2^ statistics indicated that this model accounted for: at T_3_, 8% of the variance in expressive vocabulary skills, 31% of the variance in EF and 28% of the variance in SR; and at T_2_, 37% of the variance in EF and 53% of the variance in SR. Modification indices suggested model improvement with inclusion of additional paths from T_1_ and T_2_ EF and SR to T_3_ expressive vocabulary, which were added sequentially by order of their strength and evaluated in Model 3b.

The final revised model (Figure 3B) provided better relative fit to the data: *χ*^2^(7)=20.18, *p*=0.005, CFI=0.98, RMSEA=0.06, AIC=76.18. While all paths from the initial Model 1 remained significant, there was an additional significant path from T_1_ EF to T_3_ numeracy (*β*=0.33), which rendered non-significant the paths from T_3_ EF (*β*=0.06) and SR to numeracy (*β*=0.07). This model provided better explanation of expressive vocabulary as well, *R*^2^=0.15. No further modification indices were indicated after inclusion of this path.

## Discussion

The current study elucidates longitudinal bi-directional associations between EF and SR across the transition to school period, as well as their independent and cumulative prediction of early academic skills in the first year of school. Specifically, composite indices of both EF and SR showed stability over time despite diversity in their constituent measures, yet also modest bi-directional associations with each subsequent timepoint. Both EF and SR also independently predicted early numeracy abilities and, to a lesser extent, expressive vocabulary, the prediction of which was improved by adding a direct path from initial EF levels. Together, these results point to EF and SR as related yet distinct abilities, each with direct implications for acquisition of early academic knowledge and skills, as well as indirect effects through their reciprocal influence. This contrasts with conceptions and operationalisations that treat SR and EF as effectively interchangeable (e.g., McClelland et al., 2007; Ponitz et al., 2009) and extends existing evidence of cross-sectional association longitudinally (Evers et al., 2016; Tamm and Peugh, 2019).

While previous research has established cross-sectional associations between EF and SR (Evers et al., 2016; Tamm and Peugh, 2019), the longitudinal design utilized here established ongoing bi-directional associations between EF and SR across the transition to school period. This is in line with predictions of Blair and colleagues’ bi-directional model (Blair and Ursache, 2011; Blair, 2016), which delineates volitional, cognitive EFs from temperamental and thus less overtly intentional effortful control aspects of SR (Blair et al., 2015). Despite this delineation, however, these abilities are viewed as interacting toward successful SR. Longitudinal interactions between EF and SR in the current results suggest not only that these abilities interact toward successful SR, but also appear to be mutually influential developmentally. Possible mechanisms for this include higher levels of early SR (and thereby more frequent SR success) ensuring lower levels of experienced stress and associated neurochemical release that can impede EF growth (Cerqueira et al., 2007). Similarly, with better early EF children would have a wider allostatic range at which they can self-regulate, and thus greater opportunity to practice and gain proficiency across a broader range of SR-relevant contexts, experiences, and strategies (Blair and Dennis, 2010; Blair and Raver, 2015). In contrast, children with lower EF levels may need more frequent co- or other-regulation in such situations due to their EF and SR resources being overwhelmed. The mechanisms and conditions for this reciprocal developmental influence are an important area for future study, as well as for intervention design and implementation efforts. The current findings imply that interventionists would do well to consider and target both abilities, in contrast to prominent intervention approaches that foster individual EF or SR components (e.g., Klingberg et al., 2005; Hughes and Cline, 2015); while this possibility requires further research to explicitly evaluate, examples of effective integrated interventions exist (Barnett et al., 2008).

In line with the substantial literature base showing that EF and SR predict academic skill acquisition and success when studied independently (Bull et al., 2008; McClelland et al., 2013), the current study showed that EF and SR *both* independently predicted early academic skills beyond the prediction of the other. Level of prediction did not significantly differ between the two, as constraining to equivalence their paths to early academic skills did not yield a meaningful change in model fit. It is notable that a model comprised exclusively of longitudinal indices of EF and SR composites accounted for 43% of the variance in early numeracy scores and significant, albeit comparatively less, variance in expressive vocabulary scores (15%). This pattern is consistent with Blair et al.’s (2015) finding of EF and SR measures predicting early mathematics but not letter-word knowledge. One possibility to explain this pattern is demonstration of early numeracy skills requires greater flexibility in processing and problem solving, whereas demonstration of vocabulary knowledge is more simply declarative in nature. This finding highlights likely variability in the extent and ways that EF and SR influence school readiness and success. This variability might also extend to broader conceptions of school readiness, including aspects such as peer relationships or school avoidance (Valiente et al., 2007, 2011), for which influences of EF and SR might differ.

While a direct path from EF to early academic skills was consistent with our theoretical model, an additional pathway from initial EF levels was not part of planned models, but instead emerged from modification indices. It suggested that, beyond the indirect effect of early EF on later EF and SR, initial EF levels also have a direct effect on later academic skills. This may be related to the indispensable role of EFs in learning, such as enabling the mental representation, combination, and manipulation of new with old information (working memory), updating this mental information to ensure only task-relevant information and processing (inhibition), and flexibly shifting attention with the demands of the learning situation (cognitive flexibility). Initial EF levels would thus have immediate effects on acquisition of early academic knowledge and skills, as well as cumulative indirect effects by virtue of its influence on later EF and SR. Indeed, the total effect of baseline EF was *β*=0.41 and was comprised of both direct (*β*=0.30) and indirect effects (*β*=0.11). This highlights the importance of early education and intervention efforts, which the current results suggest can have immediate, accumulating, and long-term impacts.

Future research that is designed to further investigate these associations could, for instance, shed important light on the antecedents and contexts that influence this association, as well as diversity in the outcomes they influence. For instance, a prospective longitudinal study of EF and SR trajectories and outcomes could ensure stability across measures and informants. In the current study, a reduction in stability from T_2_ to T_3_ SR could have resulted from the change in informant (i.e., preschool educator to Kindergarten teacher) and/or revision of measures comprising the composite index (i.e., inability to run PRSIST in schools given insufficient numbers of participants at each school, necessitating use of two CSBQ SR subscales alongside HTKS at T_3_). While not the ideal situation, there remained good stability over time and prediction of early academic skills, which suggests robustness of this composite approach to SR estimation and strength of its association with early academic skills.

Some additional methodological decisions also contextualise the current findings. It should be acknowledged that fixed order of task administration does introduce the potential for order effects. While such effects are possible, we had no reason to expect that the order of task administration would either influence between-construct associations or affect children’s academic readiness scores in a systematic manner. Fixed task order is common in similar studies and typically reflects a priority to maximize children’s engagement and motivation. There is also a lack of clarity about whether/which measures index EF rather than SR. This is complicated by the prevailing view that EFs are involved in SR. This is perhaps best illustrated by our adoption of HTKS. HTKS was created as a measure of behavioral SR (Ponitz et al., 2009), but at the same time is conceptualized as an EF measure in a number of recent studies due to its need to hold rules and instructions in mind, resist performing the instruction as delivered, and flexibly shifting between instructions and blocks (Liu et al., 2018; Keown et al., 2020). Our inclusion of additional measures that more clearly tap SR (e.g., CSBQ captures child behaviors such as persistence with difficult tasks; PRSIST provides ratings of whether children stay within the rules of the activity) – and combining these into a composite score that is less influenced by individual task characteristics – mitigates the impact of particular task inclusions. Indeed, modest correlations between the EF and SR indices support the perspective that the constructs generated are indeed distinct (yet related). Lastly, in Model 3 we omitted the emotional SR scale from CSBQ when creating the composite variable. This was on statistical grounds, given low inter-task correlation for the emotional SR subscale (unlike the overall SR index) with HTKS across all timepoints (*r*s up to 0.17), but particularly at T_3_ (*r*=0.07). Our data are unable to determine why this was the case, but we speculate that this is related to the cognitive and behavioral (but perhaps not emotional) demands of HTKS. In any event, this low correlation for the emotional SR subscale precluded its combination with HTKS to create a composite index. Nevertheless, stability of the SR composite over time suggests its consistency with the earlier SR composites, while its prediction of outcomes supports the predictive validity of this composite index.

The current study provides important evidence supporting a distinction between EF and SR abilities, rather than two names for one ability, yet also for their reciprocal developmental influence. This has potential implications for early childhood education and intervention efforts. That is, although there is little doubt that EF and SR can be enhanced by education and intervention (Diamond and Lee, 2011; Pandey et al., 2018), a successful theoretical model for EF and SR change has been more elusive (Hofmann et al., 2012). This may be due, in part, to the common separation of EFs and SR in studies attempting to stimulate growth in these abilities (e.g., Klingberg et al., 2005; Hughes and Cline, 2015), or the assumption that change in one will stimulate change in the other (Klingberg et al., 2005). The current results suggest that their meaningful integration in children’s everyday contexts may be a possible way forward. Indeed, there are already some examples of success with this approach (Baron et al., 2017). Further study is also needed to understand the nature and conditions under which EF and SR interact developmentally, and it is our hope that the current findings provide important insights to support this endeavour.

## Data Availability Statement

The raw data supporting the conclusions of this article will be made available by the authors, without undue reservation.

## Ethics Statement

The studies involving human participants were reviewed and approved by University of Wollongong Human Research Ethics Committee (Social Sciences). Written informed consent to participate in this study was provided by the participants’ legal guardian/next of kin.

## Author Contributions

SH conceptualized the study, secured funding for the study, oversaw data collection, led data analysis, and writing of the manuscript. EV aided in conceptualizing the study, managed data collection and entry, and contributed to drafting of the manuscript. CN-H supported design of the study, oversaw aspects of data collection, and contributed to drafting of the manuscript. MR contributed to design and selection of measures, theoretical aspects of the study, result translation, and drafting the manuscript. AC, SJ, and EM contributed to design and selection of measures and reviewing the manuscript. MM contributed to drafting the manuscript. FP contributed to design and selection of measures, critiquing and informing data analyses, and reviewing the manuscript. All authors contributed to the article and approved the submitted version.

## Funding

This work was supported by an Australian Research Council Discovery Early Career Researcher Award grant (DE170100412).

## Conflict of Interest

The authors declare that the research was conducted in the absence of any commercial or financial relationships that could be construed as a potential conflict of interest.

## Publisher’s Note

All claims expressed in this article are solely those of the authors and do not necessarily represent those of their affiliated organizations, or those of the publisher, the editors and the reviewers. Any product that may be evaluated in this article, or claim that may be made by its manufacturer, is not guaranteed or endorsed by the publisher.
